# Supplementary material for: New Fossil Evidence Suggests That Angiosperms Flourished in the Middle Jurassic
Source: Life (Basel). 2023 Mar 17;13(3):819. doi: 10.3390/life13030819 (PMC10059865; doi:10.3390/life13030819)
Supplement: Supplementary file 1 [file life-13-00819-s001.zip › Supplementary Material.docx]

New fossil evidence suggests that angiosperms flourished in the Middle Jurassic

**Lei Han^1^, Ya Zhao^2^, Ming Zhao^2^, Jie Sun^3^, Bainian Sun^1,4^** **^*^, Xin Wang^4 *^**

^1^ Key Laboratory of Minerals Resources in Western China (Gansu Province), School of Earth Sciences, Lanzhou University, Lanzhou 730000, China

^2^ Ningxia Geological Museum, 301 Eastern People’s Square Street, Yinchuan 750000, China

^3^ Shaanxi Key Laboratory of Early Life and Environments, State Key Laboratory of Continental Dynamics, Department of Geology, Northwest University, Xi’an 710069, China

^4^ State Key Laboratory of Palaeobiology and Stratigraphy, Nanjing Institute of Geology and Palaeontology and CAS Center for Excellence in Life and Paleoenvironment, Chinese Academy of Sciences, Nanjing 210008, China

***** Correspondence: xinwang@nigpas.ac.cn; bnsun@lzu.edu.cn


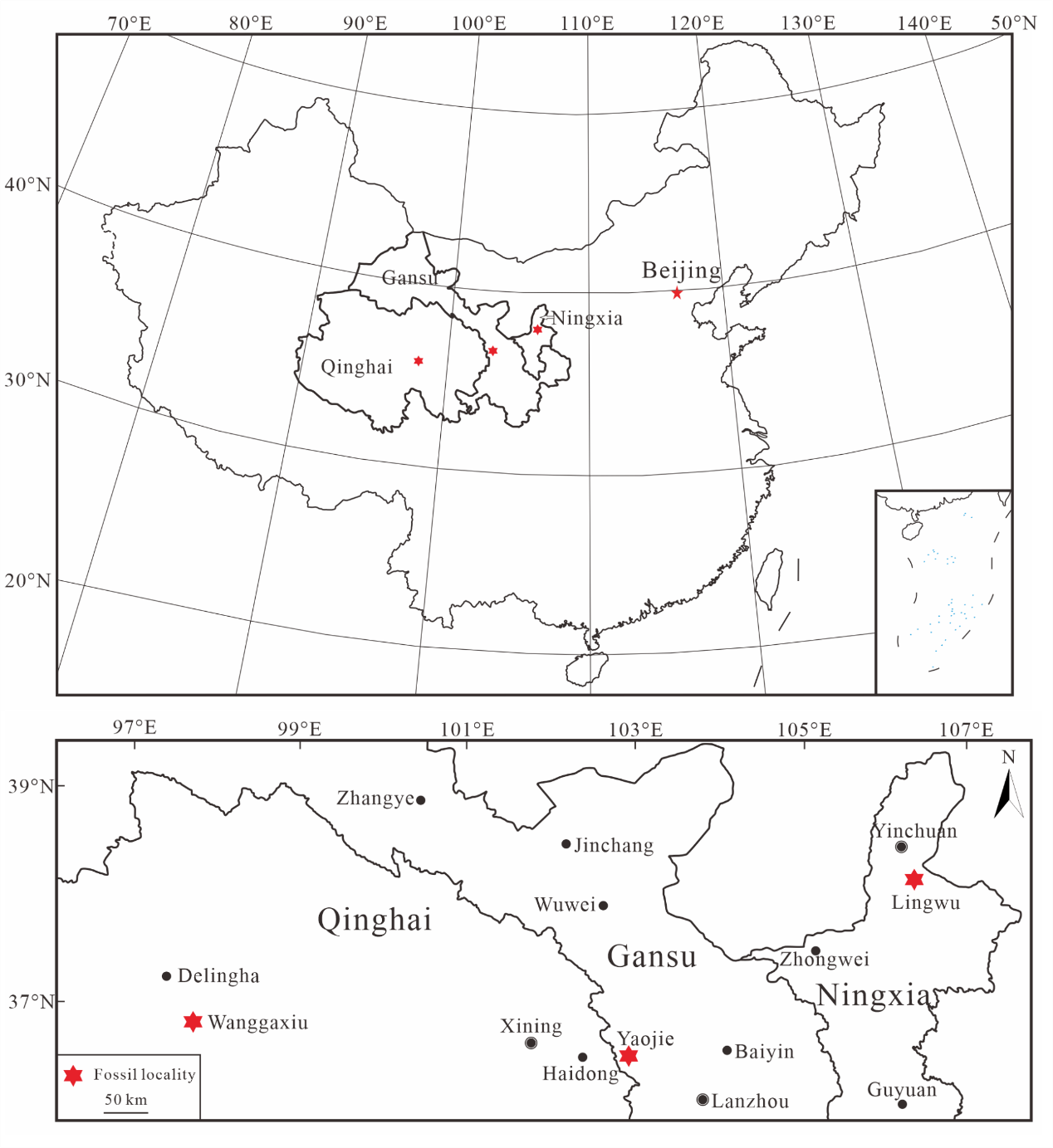


**Figure S1.** Geographical positions of the fossil localities.


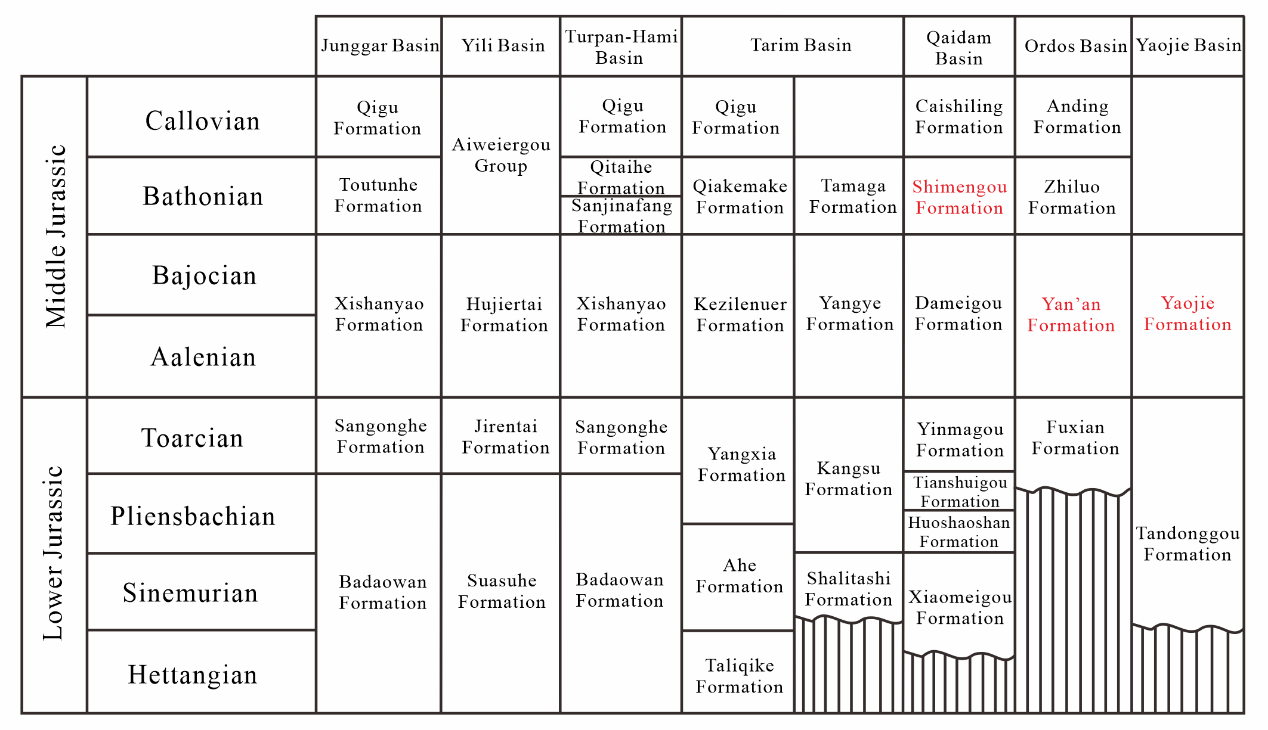


**Figure S2.** Stratigraphical correlation of the Jurassic coal-bearing strata between the Yaojie Basin and adjacent regions in Northwest China (modified from Zhang et al. (1998)).

**
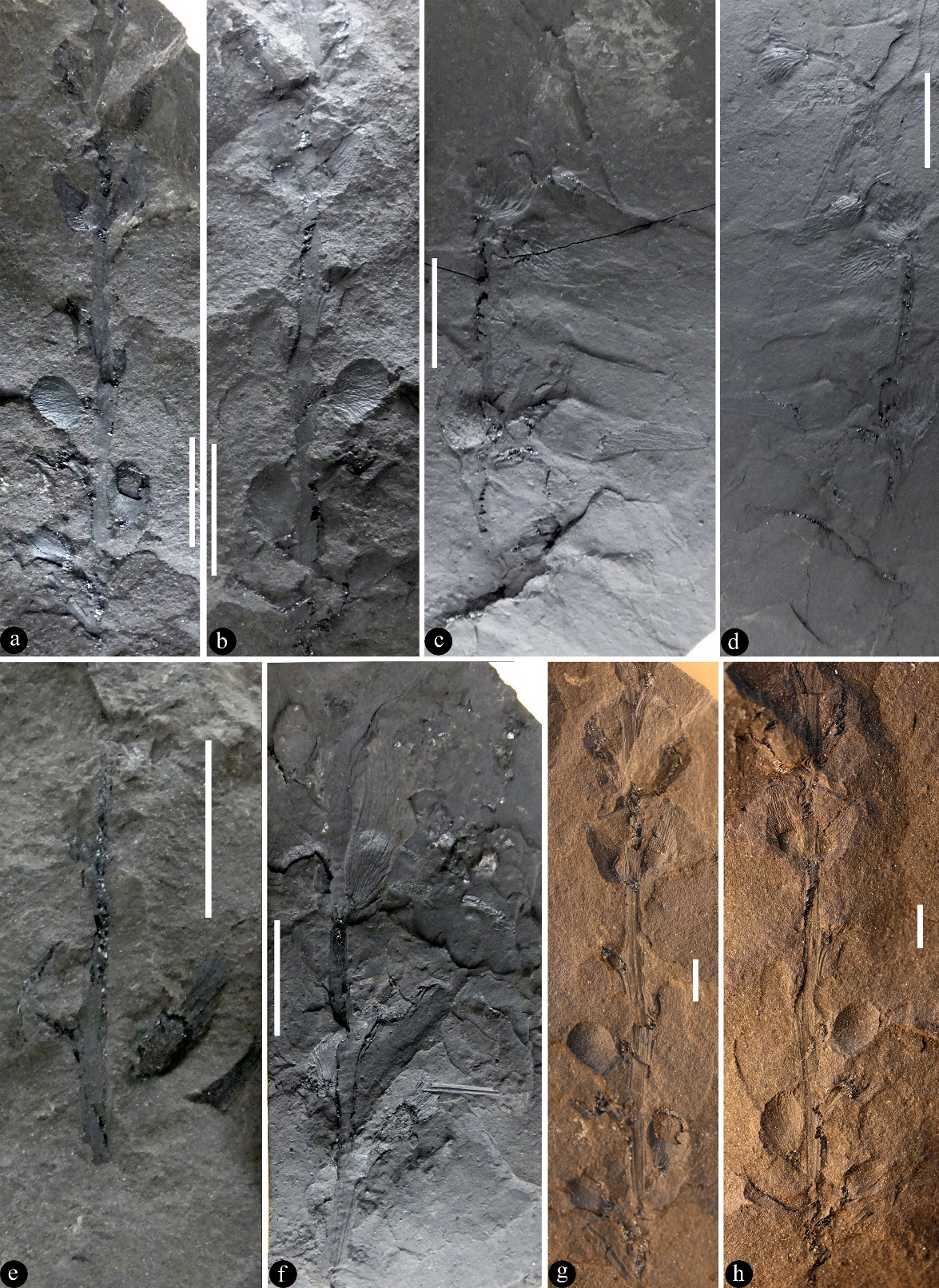
**

**Figure S3.** Infructescences of *Qingganninginfructus* *formosa* gen. et sp. nov., preserved in various ways, each with multiple fruits spirally arranged along its rachis. (**a,b**). (LZP-2018-07A, LZP-2018-07B), **(c,d)**. (LZP-2018-01A, LZP-2018-01B), (**g,h**). (LZP-2018-11A, LZP-2018-11B) are of parts and counterparts, respectively. (**e**). LZP-2018-08. (**f**) LZP-2018-10. Scale bar = 5 mm.


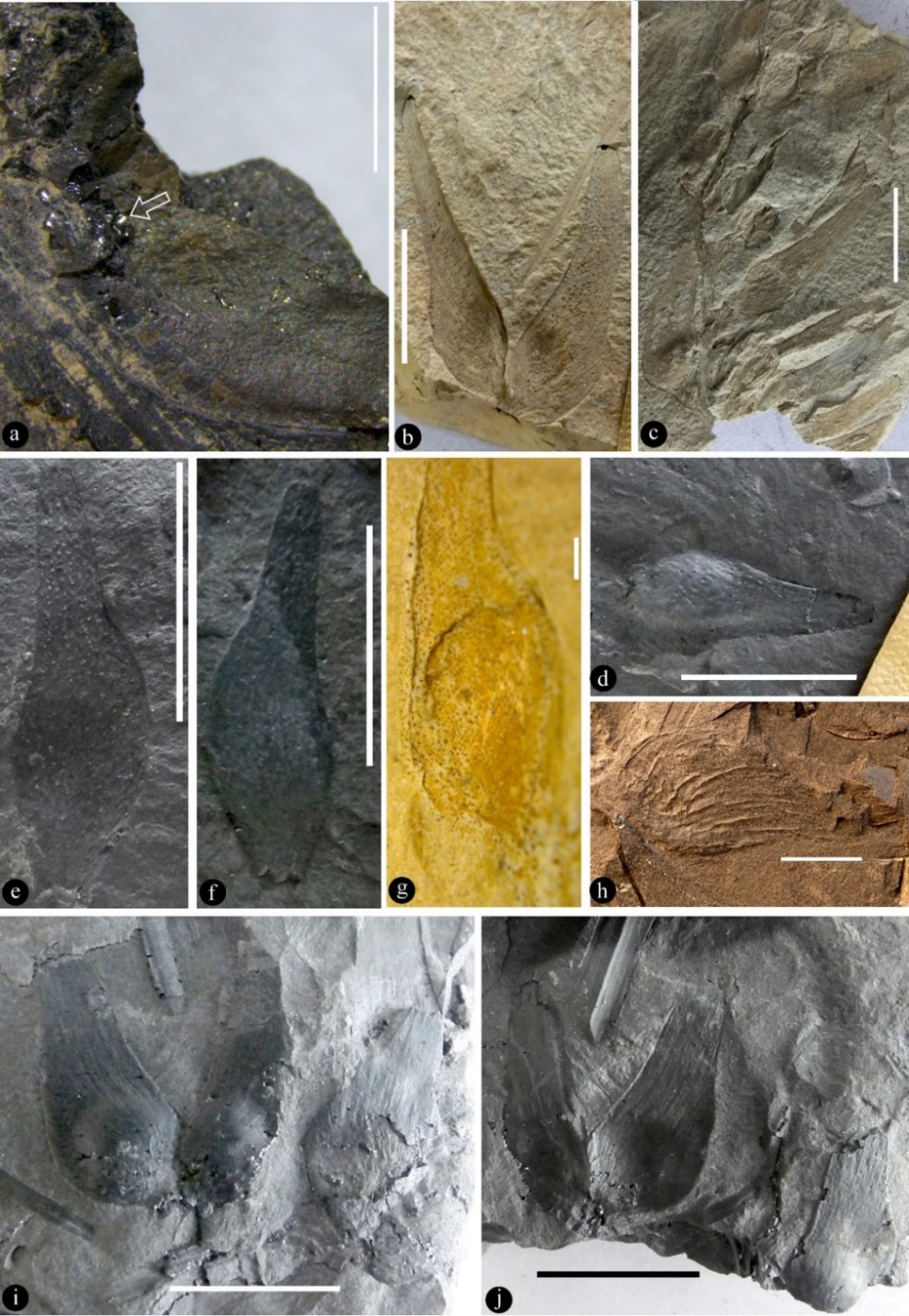


**Figure S4.** Fruits of *Qingganninginfructus* *formosa* gen. et sp. nov. (**a**). A broken fruit with residue of a ovule (arrow) with the ovule’s side view (in the direction of the arrow) exhibited in Figure 1d. LZP-2018-09. Scale bar = 2 mm. (**b**). A pair of fruits, showing their outlines and depressions left by basal *in situ* ovules in the fruits. LZP-1984-59. Scale bar = 5 mm. (**c**). Several fruits in an infructescence. LZP-1984-57. Scale bar = 5 mm. (**d,f**). Three fruits, showing their outlines. LZP-2018-05B, LZP-2018-06A, LZP-2018-06B. Scale bar = 5 mm. (**g**). A broken fruit showing a basal ovule *in situ*, from the specimen shown in Figure 1b. Scale bar = 1 mm. (**h**). A fruit with obvious longitudinal striations, enlarged from Figure 1a. Scale bar = 5 mm. (**i,j**). Part and counterpart showing paired coalified fruits with longitudinal striations and a basal ovule *in situ*. The left fruit in Figure S4i is detailed in Figure 3f-i. LZP-2018-03B, LZP-2018-03A. Scale bar = 5 mm.


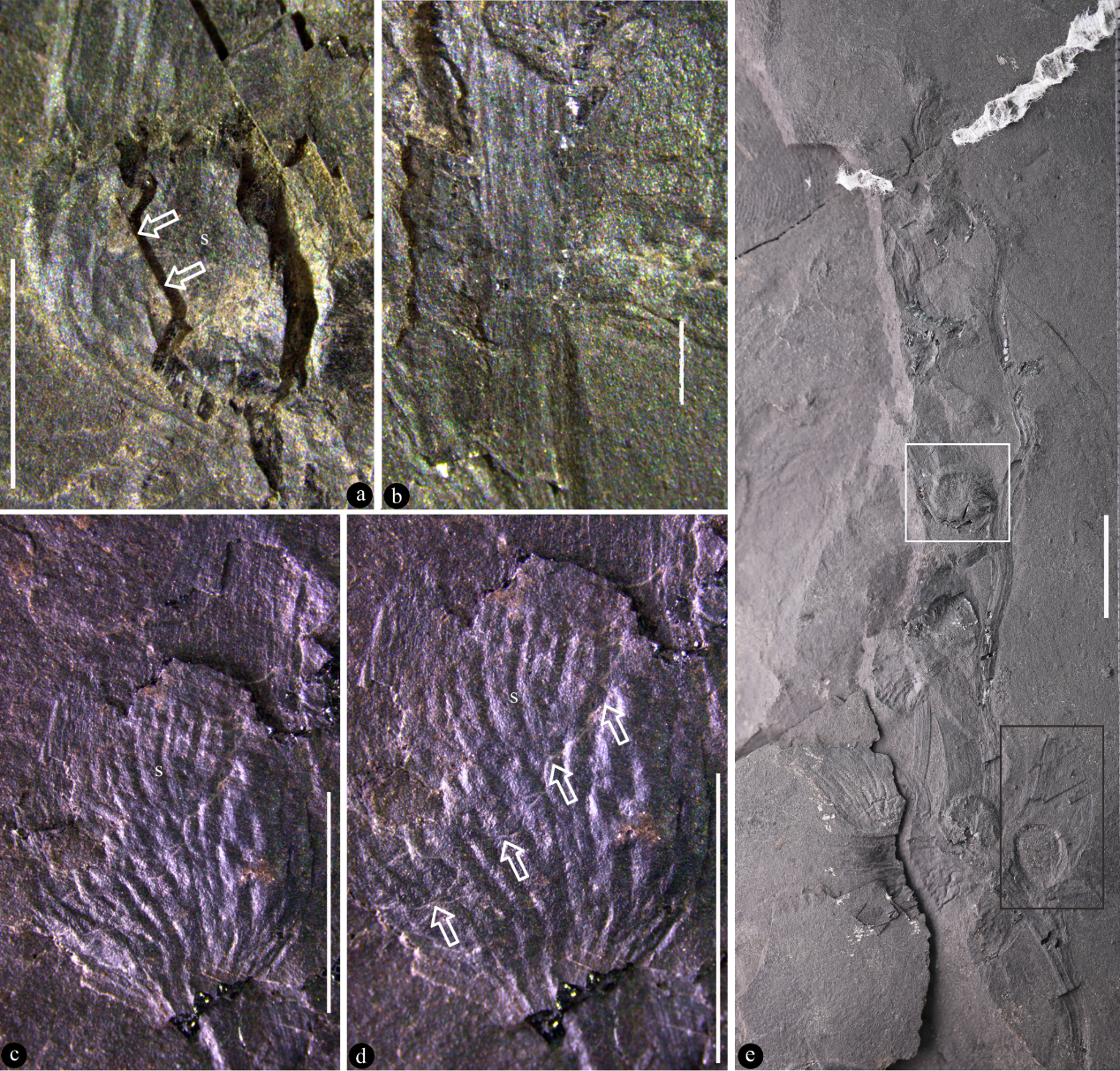


**Figure S5.** Fruits with *in situ* ovules of *Qingganninginfructus* *formosa* gen. et sp. nov. (**a**). A broken fruit showing a partially-covered ovule in the fruit. Scale bar = 5 mm. (**b**). Detailed view of the rachis of the infructescence whose fruits are shown in Figure 1e-h, Figure S5a, Figure S6a, d-e. Scale bar = 2 mm. (**c**). The topmost fruit in Figure S3c, showing ovule (s) partially covered in the fruit. Scale bar = 5 mm. (**d**). Margin of the broken fruit wall (arrows) covering the ovule (s), enlarged from Figure S5c. Scale bar = 5 mm. (**e**). The counterpart of the specimen shown in Figure 1a, showing an infructescence including several fruits spirally arranged along the its rachis. LZP-2018-02B. Scale bar = 1 mm.


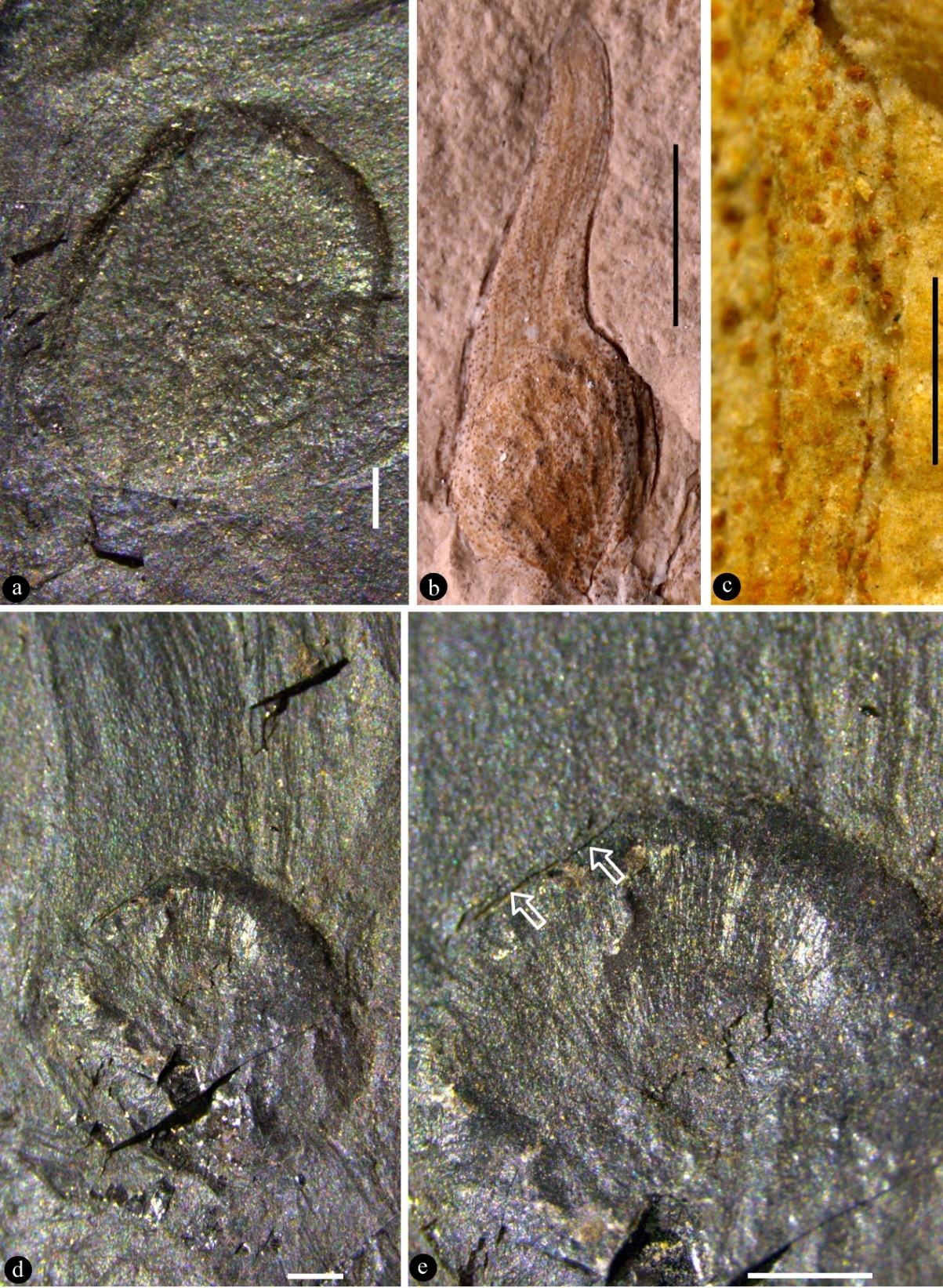


**Figure S6.** Fruits and ovules of *Qingganninginfructus* *formosa* gen. et sp. nov. (**a**). An *in situ* ovule in the broken fruit shown in Figure 1g (rectangle). Note its smooth margin in the upper part suggestive of a basal connection. Scale bar = 1 mm. (**b**). The topmost fruit in the infructescence shown in Figure 1b, with an *in situ* ovule. Scale bar = 5 mm. (**c**). Detailed view of a layer (ovule coat) surrounding the ovule in Figure S6b. Scale bar = 1 mm. (**d**). A broken fruit with an *in situ* ovule in Figure S5e. Scale bar = 1 mm. (**e**). Detailed view of the *in situ* ovule in Figure S6d, with the radiating sculpture and the surrounding fruit wall (arrows). Scale bar = 1 mm.


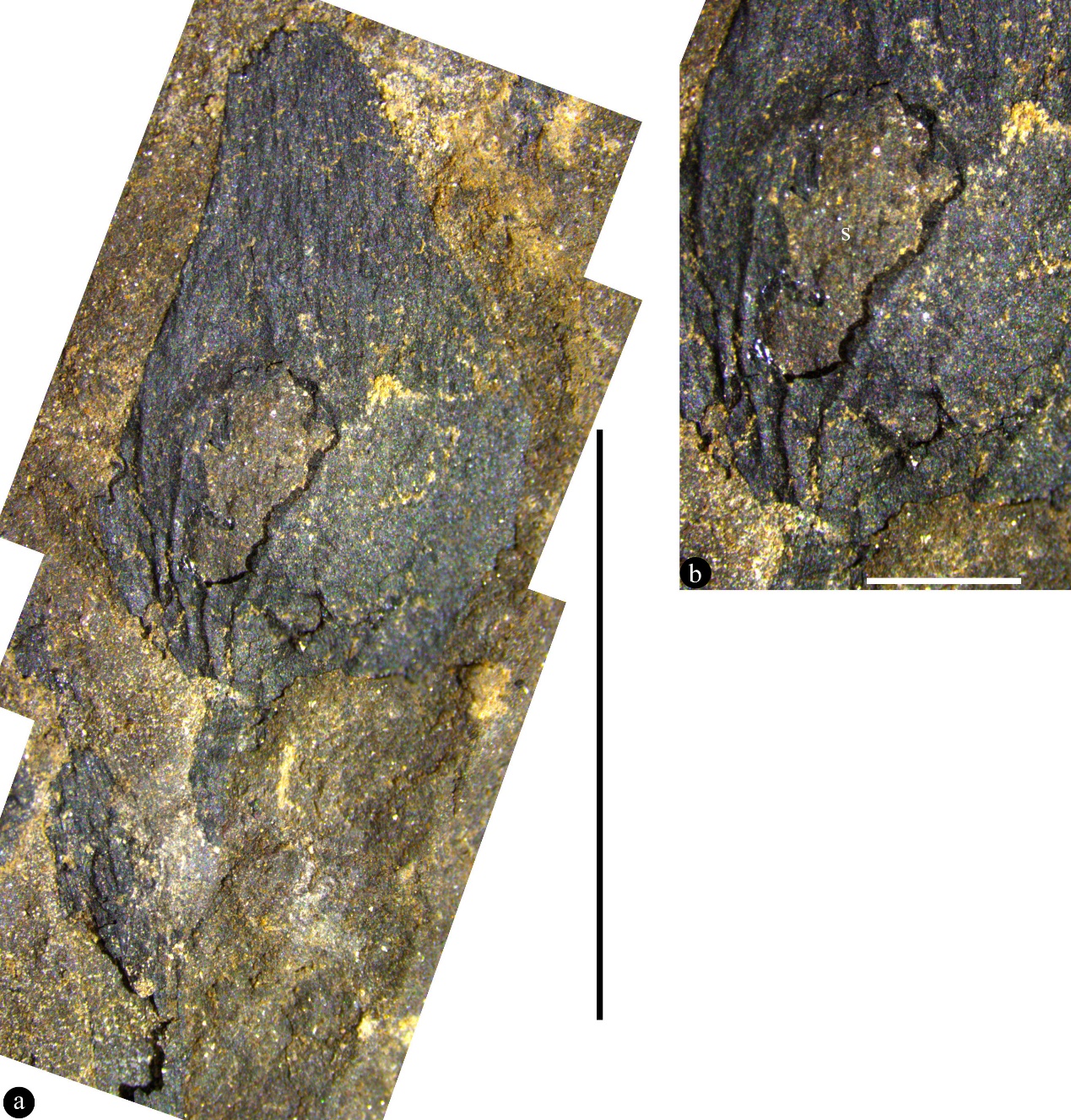


**Figure S7.** Infructescences of *Qingganninginfructus* *formosa* gen. et sp. nov. (**a**). A broken fruit connected to its infructescence rachis (lower left), showing its round-triangular shape and pedicel. LZP-2018-04. Scale bar = 5 mm. (**b**). Detailed view of the fruit in Figure S7a, showing the broken fruit wall and exposed *in situ* ovule (s). Scale bar = 1 mm.

**
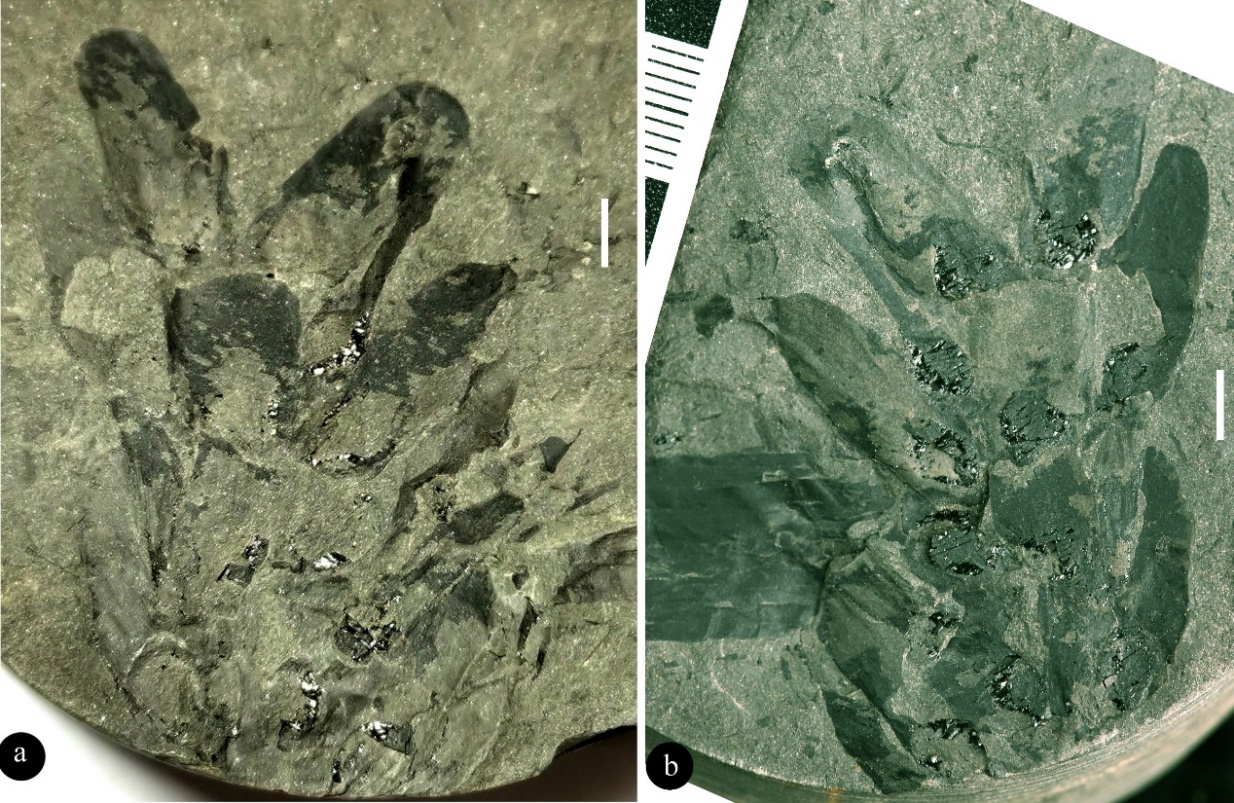
**

**Figure S8.** Infructescences of *Qingganninginfructus* sp. (**a**). General view of the infructescence of *Qingganninginfructus* sp., showing the wide round tips of the fruits and dark shiny ovules within fruits. GSW615. Scale bar = 5 mm. (**b**). Counterpart of the specimen shown in Figure S8a. GSW870. Scale bar = 5 mm.

**Video S1** Video of the infructescence shown in Figure 2.

**Video S2** Video of the sheathed fruit pedicel shown in Figure 2b.

**Video S3** Video of the micropyle and funiculus shown in Figure 3e.

**References**

1. Zhang, H.; Li, H.T.; Xiong, C.W.; Zhang, H.; Wang, Y.D.; He, Z.L.; Lin, G.M.; Sun, B.N. *Jurassic coaliferous strata and coal accumulation in Northwest China*. Geological Publishing House: Beijing, China, 1998.
